# Supplementary material for: Architecture and evolution of semantic networks in mathematics texts
Source: Proc Math Phys Eng Sci. 2020 Jul 29;476(2239):20190741. doi: 10.1098/rspa.2019.0741 (PMC7426037; doi:10.1098/rspa.2019.0741)
Supplement: Supplementary Material for "Architecture and evolution of semantic networks in mathematics texts" [file rspa20190741supp1.pdf]

Supplementary Material for "Architecture and evolution of semantic networks in mathematics texts"

Nicolas H. Christianson, Ann Sizemore Blevins, Danielle S. Bassett

Summary of Supplementary Material

In this supplementary document, we provide supplemental methods, followed by supplemental results.

Supplementary Methods

Textbooks used

The ten textbooks used in our study [1, 2, 3, 4, 5, 6, 7, 8, 9, 10] have publication dates ranging from 1967 to 2018. The set also includes two texts that were translated from a different language, and two texts that are made available online for free use.

Considerations for concept extraction

In order to construct a semantic network, it is first necessary to choose which concepts should comprise the nodes of that network. Much previous work has considered all or most of the individual words in a text as the network nodes [11, 12]; we avoid this assumption so that we may consider, further than individual words, higher-level concepts that may be presented in multi-word phrases. Another choice of nodes could be the topics present in the index of a text, if an index is included. We also choose not to use this method, as we seek to determine and extract the concepts from the text's exposition via some more intrinsic metric of conceptual significance. This choice was motivated by an interest in examining the semantic networks of concepts that the text poses as significant, rather than simply those of concepts which the author deems significant. Thus, via this paradigm of intrinsic conceptual significance, we aim to emulate human readers in their assessment of the significance of concepts. In choosing a methodology of extracting concepts from the texts for use as the networks' nodes, we sought to find a method that would maximize the number of extracted mathematical concepts while minimizing the number of extracted words and phrases that are not mathematics related. We also sought a method that would be extensible to domains of knowledge and exposition aside from mathematics, so that our whole methodology can be extended to the analysis of general textual exposition. These considerations led to our development of the modified RAKE algorithm.

Implementation details for our concept extraction methodology

In our code, we use the python-rake implementation of RAKE (*https://github.com/fabianvf/python-rake*); as a stop word list, we use the modified Ranks NL Long stop word list we discuss in the main text, from which we remove the word "value",which plays an important role in linear algebra phrases such as "singular value decomposition". We also add to this stop list our placeholder words "#", "VAR", and "-pron-" (the pronoun placeholder output by the spaCy lemmatizer), as well as certain words used extensively in mathematics exposition that do not convey mathematical content, in an effort to ensure that our keyphrases might better reflect a set of meaningful mathematical concepts (see Supplementary Table S1). We also prune the candidate pool by specifying that keywords must be comprised of at least 3 characters and must occur at least 5 times within the text, and keyphrases can be no more than 4 words long. Given these specifications, RAKE generates a set of candidate keyphrases and their associated scores, which we modify with the addition of our extra Brown frequency term. We then clean the candidate keyphrases by removing any of

examples	counterexample	text	texts	undergraduate	chapter
definition	notation	proof	exercise	result	

Table S1: Common words in mathematical exposition that we add to the stop word list for concept extraction

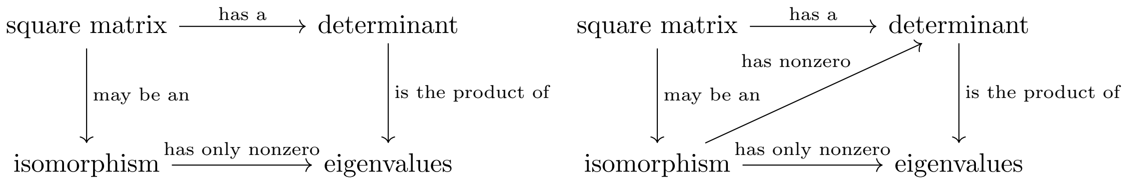

(a) Unfilled knowledge gap in the network

(b) Knowledge gap is filled

Figure S1: A simple example of a semantic network comprised of linear algebra concepts. *(a)* The lack of connection between "square matrix" and "eigenvalues" or between "isomorphism" and "determinant" indicates the presence of a knowledge gap. *(b)* The knowledge gap is extinguished by the addition of the relationship between "isomorphism" and "determinant," thus ensuring that all concepts' neighbors are also neighbors themselves.

the numerical, variable, or pronoun placeholders; after this cleaning, if there are any duplicate candidates, we give the keyphrase in question the highest score from all duplicates. We choose to keep the top-scoring half of candidate keyphrases, since this threshold appears to include most phrases one might expect to represent significant linear algebra concepts in each text; thus we take the top half of scored keyphrases to be the concept set for each text, which we refer to as the *index list*. This threshold of one-half is similar to thresholds used in other work, such as the threshold of one-third in RAKE [13] and Textrank [14]. However, no choice of threshold will perfectly include all relevant concepts and omit irrelevant words.

Considerations for network construction

Once we have determined a set of concepts to use as the nodes of a text's semantic network, we then wish to form the semantic network of those concepts and their relationships, as provided by the text's exposition. Certain approaches to semantic network construction seek to determine not only whether two entities are related, but also the semantic nature of the relationship between the entities in question. Fig. S1 gives an example of such an annotated semantic network, in which each relation has a meaningful label. Such semantic parsing techniques to generate semantic networks have been applied to scientific texts in several cases [15, 16], but they generally require involved syntactic parsing rules or data annotation. We did not use these approaches, as the messy nature of the text-converted mathematics textbooks – with embedded variables, formulas, and symbols sometimes interjecting sentences – likely would have interfered with effective inference of semantic relationships. Instead, we use a method of extracting concept relationships that is more resistant to such noise: co-occurrence frequency [17]. Co-occurrence is a notion specifying the degree to which words or phrases tend to occur nearby each other in either a text or a set of texts. Statistical metrics based on co-occurrence have been studied extensively in the field of computational linguistics as a measure of the semantic relatedness of words or phrases [18, 19, 20]. Because we are interested in relationships between concepts which are not purely linguistic in nature, and since many of our extracted concepts are multiple-word phrases, we choose to calculate co-occurrence on the sentence level; this level of granularity will also ensure that phrases in the same sentence, yet separated by a string of math variables, will be inferred to be related.

Null models

Here, we describe in more detail the construction and role of each null model we employ in our work. We begin with the data-level null models: for both the total network and the expositional filtration, we wish to

determine the extent to which our results might simply be reflective of the topology one would expect from the growing "semantic network" generated by computing the co-occurrence of a random set of words in our texts. To this end, we employ a *random index* null model, in which we select a random set of index terms of equal size to the original index list, drawn without replacement from the set of words comprising each text (not including the augmented stop word list we used for RAKE extraction). We use this random index list as our set of "concepts" for calculating each text's co-occurrence, yielding both a final weighted network, as well as an expositional filtration, allowing this null to be used both in the comparison of meso-scale structure and development, as well as of persistent homology. Note, however, that we may interpret the random index null model in a different way: that is, since the random index set excludes any stop words, it must be comprised of meaningful words. Thus, the random index model can be viewed as conveying a semantic network – not the network that the book intends to convey, but a semantic network nonetheless that may very well include some mathematically meaningful concepts.

We further seek to establish the extent to which our results on topological development of the networks are dependent on the order in which relationships are introduced within the texts. We therefore employ a *random sentence order* null model, in which for each text, we randomly permute that text's sentences, and use the original set of index terms to calculate co-occurrence. This null model yields the same total network, since the index set is the same and the same sentences are present, and thus the same sentence-level co-occurrences will occur; however, the filtration it yields will differ in the order of edge introduction, thus enabling us to study how the meso-scale and topological development of the network differs based on differing sentence order.

The remainder of our null models are projected network-level nulls. To evaluate the extent to which the results we observe for the core-periphery and community structure of the empirical networks would be expected from a random network with a similar joint distribution of node degrees and weights, we use the *continuous configuration model* [21]. This model is an extension of the configuration model for random graph generation, and seeks to preserve the expected degree of each node, as well as the expected strength of the node, where a node's strength is the sum of the weights of the edges it participates in. Specifically, if d_u and s_u give the degree and strength, respectively, of a node u, and d_T and s_T are the sum of all node degrees and strengths, respectively, then given some graph with node set $[n]$, for any two nodes $u, v \in [n]$, we define $d_{uv} = \frac{d_u d_v}{d_T}$ and $s_{uv} = \frac{s_u s_v}{s_T}$, as well as $\{P_{uv}\}$ as some family of probability distributions with mean one. Then to generate a graph using the continuous configuration model, we iterate through all possible pairs of nodes u, v, introducing an edge between u and v with probability d_{uv}; if an edge is introduced, then the edge is given weight $w_{uv} = \frac{s_{uv}}{d_{uv}} \xi_{uv}$ where the normalized weight random variable $\xi_{uv} \sim P_{uv}$. For the sake of simplicity, we assume that all distributions P_{uv} are identical, so that all $\xi_{uv} \overset{iid}{\sim} P$; we discuss our fitting of the distribution P for each network in the supplement.

To examine how our results on persistent homology differ from a model of exposition in which connections are drawn completely at random – that is, with a filtration of the empirical total network that adds edges randomly – we employ the *random edge* null model. In this model, edges present in the empirical total network are introduced in a random order, and nodes are introduced immediately preceding their first inclusion in an added edge. Next, to determine how our persistent homology results differ from a model of exposition in which concepts are iteratively introduced and connected to all already-introduced concepts, we examine a *node-ordered filtration* [22, 23]. In this null model, nodes are added by order of introduction in the text; if multiple nodes were originally added in a single sentence, then those nodes will be added to the node-ordered model in a random order. After each node is added to the null, all edges between it and previously-added nodes that are present in the total network are added in a random order.

Finally, we must consider a caveat for the filtration null models: in particular, while the original expositional filtration, the random index null, and the random sentence order null have some intrinsic sense of "time" of introduction due to the presence of the sentence structure of the text, the latter two null models do not, as they introduce nodes and edges one at a time. As such, in order to meaningfully compare persistence barcodes amongst all these models, we must "unfurl" the expositional filtrations of the real network and the random index networks. To this end, we introduce the one-at-a-time (OAAT) filtration process; this methodology takes a filtration in which multiple nodes and edges might be introduced in single sentences, such as the expositional filtration of a text, and transforms it so that only a single node or edge is added at each step in the filtration. Specifically, for each sentence, the OAAT process examines what nodes and

edges are added to the network in that sentence; if multiple nodes are added, then they are added first, one at a time, in a random order; then edges are added, one at a time, in a random order. For our empirical expositional network, we compute 100 instantiations of this OAAT filtration in order to account for stochasticity in the random ordering (we do not do this for each random index or sentence order filtrations, since we already compute 100 distinct such graphs). With this method, we may examine the topological development that occurs not just over the course of the text with a sentence-level granularity, but also on a sub-sentence scale.

There are certain tradeoffs we make in using the OAAT filtration for our expositional filtrations. In particular, we lose the direct relationship of cavity persistence length to "time", or sentence duration throughout the text, since we instead simply introduce one node or edge at each "timestep" in the OAAT filtration. However, long cycles should still tend to be long, under the assumption that there is relatively consistent introduction of nodes and edges throughout the texts. Furthermore, this "unfurling" of the expositional filtration gives us the ability to do a tête-à-tête comparison of our latter two null models to the expositional filtrations. These two nulls have no built-in notion of time, and introduce a single node or edge at each step of their filtration; as such, putting our expositional filtrations on equal footing makes the qualitative and quantitative comparison of the persistent homologies of these filtrations more direct.

Supplementary Results

Estimating the normalized weight distributions for the continuous configuration model

The parametrization of the continuous configuration null model for weighted undirected graphs rests upon the choice of a family of probability distributions P_{uv} that specifies the distribution of the possible "normed weight" values for each edge connecting nodes u and v in a network's node set. Specifically, where d_u and s_u are the degree and strength, respectively, of a node u, and d_T and s_T are the sum of degrees and strengths respectively over all nodes in a network, and $d_{uv} = \frac{d_u d_v}{d_T}$ and $s_{uv} = \frac{s_u s_v}{s_T}$ give a normalized view of to what extent two nodes are both high (or low) in degree or strength, then the continuous configuration model assumes that the weight of an edge between two nodes u and v, if such an edge exists, will be

$$w_{uv} = \frac{s_{uv}}{d_{uv}} \xi_{uv}$$

where $\xi_{uv} \sim P_{uv}$, some probability distribution on what we call the "normalized weight" of an edge. In our work, for the sake of simplicity, we make the assumption that all normalized weight distributions are the same distribution P. With this assumption, we may choose a parametrization of P and fit this distribution on the empirical normalized weights of all edges in a given network. In particular, if the empirical edge weights are given as $\hat{w}_{uv}$ for all u, v in the set of edges, then the empirical normalized weights are simply given by $\frac{\hat{w}_{uv} d_{uv}}{s_{uv}}$.

Once we have the normalized weights, we may choose a parametrization. Because the normalized weights of a network are positive and not restricted to the integers, we attempted maximum likelihood fits of a number of continuous probability distributions with support on the positive real line on each of the networks' normalized weights. Specifically, we focused on long-tailed distributions: the Pareto, Log-normal, Lévy, Burr, Fisk, Log-gamma, Log-Laplace, and power-law distributions. We also calculated the Kolmogorov-Smirnov (K-S) statistic D of each best-fit distribution in order to determine how well the distribution fit the empirical normalized weight data. Distributions were fit and K-S statistics were calculated in Python with the SciPy library, version 1.1.0 [24]. In all networks, the K-S statistic was quite low ($D < 0.025$) with p-values all significantly greater than 0.05, indicating good fit between the empirical and best-fit distributions, or insufficient evidence to reject the null hypothesis that the empirical normalized weight distribution and the best-fit distribution are identical. The best-fits and statistics for each text's network are reported in Table S2.

Concepts that appear in more than half the semantic networks' cores

See Table S3.

Text	Best-fit distribution	K-S statistic	K-S p-value
Treil	Burr	0.0163	0.303
Axler	Burr	0.00997	0.795
Edwards	Log-normal	0.0232	0.195
Lang	Log-normal	0.0140	0.687
Petersen	Burr	0.0165	0.174
Robbiano	Fisk	0.00964	0.847
Bretscher	Burr	0.00870	0.758
Greub	Burr	0.0146	0.436
Hefferson	Burr	0.00696	0.910
Strang	Burr	0.00762	0.759

Table S2: Best-fit distributions and corresponding K-S statistics and p-values for the normalized weight distribution of each text.

Concept	Frequency in cores
multiplication	8
vector space	7
scalar	7
vector	8
inverse	8
matrix	9
polynomial	7
coefficient	8
linear transformation	6
linear	8
linearly independent	9
diagonal	9
theorem	9
projection	6
orthogonal	9
invertible	6
subspace	9
determinant	9
diagonal matrix	6
eigenvalue	9
eigenvector	8
orthonormal	7
orthonormal basis	6
equation	7
symmetric	6

Table S3: Concepts that occur in more than half of the texts' cores.

Community	Example concepts
1	commutative, associative, dual space, dual map, duality, column rank, row rank
3	finite dimensional subspace, orthogonal, orthogonal complement
4	inverse, additive inverse, additive identity
6	null space, injective, surjective, isomorphism, invertibility, identity map
7	induction hypothesis, division algorithm, factorization
8	linearly dependent, linear combination, orthonormal list, gramschmidt procedure
9	euclidean inner product, dot product, continuous real value[d] function, derivative
10	positive operator, adjoint operator, complexification, complex spectral theorem
11	transpose, permutation, determinant, square matrix

Table S4: Example concepts present within communities in the Axler periphery.

Example concepts in the Axler periphery communities

See Table S4.

Development of the meso-scale core-periphery and community structures

Similar to our analysis of the development of each text's core and periphery, we further wish to examine the development of the community structure in the semantic networks through the addition of edges between particular groups over the course of exposition. Specifically, we consider four edge types: 'core-periphery' edges, or those connecting a core node with a periphery node; 'intra-core' edges, connecting two core nodes; 'inter-periphery' edges, connecting nodes in two different periphery communities; and 'intra-community' edges, connecting two nodes in the same periphery community. We examine the relative introduction of each group of edge types by calculating, at each point in the texts' expositions, what fraction of edges in a particular group have been introduced. We show in Fig. S2 the mean ± 2 standard deviations of these group introduction curves across all texts; for the intra-community curves, we plot two examples: one of an early-introduced community, which attains a value near 1 indicating near-completion relatively quickly, and one of a late-introduced community, which takes longer to be fully developed, and remains closer to 0 throughout much of the text.

Note that while the core-periphery, inter-community, and intra-core edge sets appear to be introduced steadily, showing little deviation from the diagonal $y = x$, which reflects constant introduction over time, the early and late intra-community examples shown have significant variability and deviate greatly from such a rule of constant introduction. We may quantify this behavior of deviation from constant introduction with the Kolmogorov-Smirnov (K-S) distance: in particular, for any of the edge group development curves $c(\cdot)$, we examine its K-S distance, or greatest vertical distance, to the line $y = x$ on the interval $(0, 1)$:

$$\text{K-S}(c) = \max_{t\in(0,1)} |c(t) - t|.$$

Note that we chose our early- and late-introduced communities in Fig. S2 as those communities with the most positive and negative values of $c(t) - t$ on the interval $(0, 1)$, respectively. We plot the resulting K-S metrics for each edge group type across all texts and corresponding null models in Fig. S2b-e. We find relative consistency across texts in relatively low K-S values for the intra-core, core-periphery, and inter-community groups, and notably, in many cases it appears as though the actual texts exhibit lower K-S values, and thus more constancy in edge introduction in these groups, than the bulk of the random index and random sentence order graphs (Fig. S2b-d). Notably, we also observe that while many of the texts exhibit lower mean intra-community K-S values than the bulk of the random index networks, they also generally lie well above the distribution of values for the random sentence order null graphs. Thus, this pattern of findings suggests that while the texts generally exhibit significant variability in when intra-community edges are introduced during the exposition, the reordering of sentences that occurs in the random sentence order model disrupts this variability, causing a community's edges to, on average, be introduced in a more distributed fashion over the course of the reordered 'exposition'. In turn, these findings suggest that the periphery communities extracted

Correlate	Spearman corr. coef.	Spearman p-value	Pearson corr. coef.	Pearson p-value
NACL, dim. 0	0.143	0.760	0.466	0.291
NACL, dim. 1	0.036	0.939	-0.334	0.464
NACL, dim. 2	0.0	1.0	-0.145	0.757
Avg. NACL	0.071	0.879	-0.187	0.689
OAAT NACL, dim. 0	-0.857	0.0137	-0.821	0.0237
OAAT NACL, dim. 1	-0.500	0.253	-0.575	0.177
OAAT NACL, dim. 2	-0.893	0.00681	-0.846	0.0163
Avg. OAAT NACL	-0.821	0.0234	-0.828	0.0213

Table S5: Spearman and Pearson correlation coefficients and p-values for Goodreads ratings and normalized average cycle lifetimes (NACLs).

from the empirical networks do indeed represent distinct groups of related concepts that are localized in their position in text, as we might expect from a chapter focusing on a particular topic (Fig. S2e).

Barcodes and Betti curves for all texts and null models

For the barcodes and Betti curves of the sentence-granularity text filtration, random index model, and random sentence order model, see Figs. S3, S4. For barcodes and Betti curves of the OAAT text filtration and all null ensembles, see Figs. S5, S6.

Normalized average cycle lifetime for texts and all null ensembles

For normalized average cycle lifetimes of the sentence-granularity filtrations for the empirical texts, random index model, and random sentence order model, see Fig. S7. For the normalized average lifetimes of the OAAT filtrations for the empirical texts and all null models, see Fig. S8.

Extended correlation analysis

In the main text, we report results of a brief exploratory analysis assessing the relationship between structural features of exposition and community ratings of the textbooks from which the expositions are taken. Here, we provide the complete statistics for the Spearman and Pearson correlations between average rating on Goodreads and normalized average cycle lifetime (NACL) in Table S5. We also note that while we consider average rating across editions, the default rating presented by Goodreads for textbooks, this metric should reasonably approximate the rating of each specific text edition we consider, since textbook editions tend to be similar.

We furthermore examine additional correlations between text features, both structural and otherwise, in Fig. S9, with associated p-values in Fig. S10. Notably, while we observe correlations between average and dimension-2 OAAT NACL and both number of sentences and node count of each text, neither of the latter structural features are significantly correlated with the average text rating. Furthermore, though the number of ratings for each text is highly variable (Table S6), we find that this number does not significantly correlate with text rating (Spearman $\rho = 0.464$, $p = 0.294$). Finally, we find that both dimension-0 and average OAAT NACL are negatively correlated with the frequency of the word “proof” in the texts’ sentences (Spearman $\rho = -0.782$, $p = 0.00755$ and $\rho = -0.697$, $p = 0.0251$, respectively), suggesting that theoretically-focused linear algebra texts might minimize the extent to which knowledge gaps are created and persist, compared to more applied texts. All correlations and p-values reported here and in the corresponding section of the main text were calculated using the Pingouin Python library, version 0.2.8 [25].

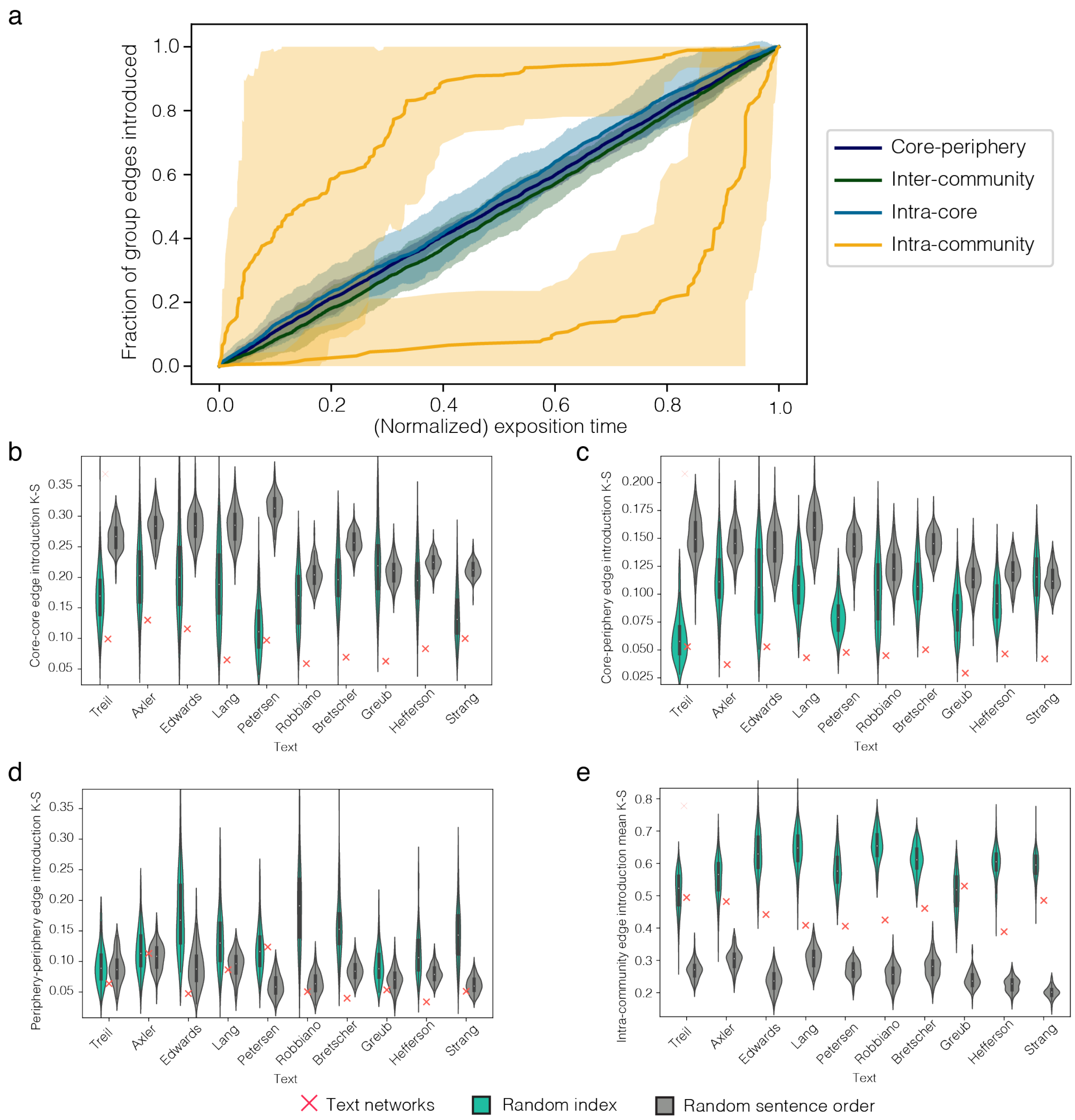

Figure S2: Community development curves across texts, and associated K-S distance between community development curve types and the line $y = x$ across all texts and null ensembles. *(a)* Mean $\pm$ 2 standard deviations of community development curves (fraction of edges within a particular group present at a particular normalized time in the exposition) across all texts, *(b)* K-S distances for the core-core edge introduction curve, *(c)* K-S distances for the core-periphery edge introduction curve, *(d)* K-S distances for the periphery-periphery edge introduction curve, and *(e)* mean K-S distances across intra-community edge introduction curves.

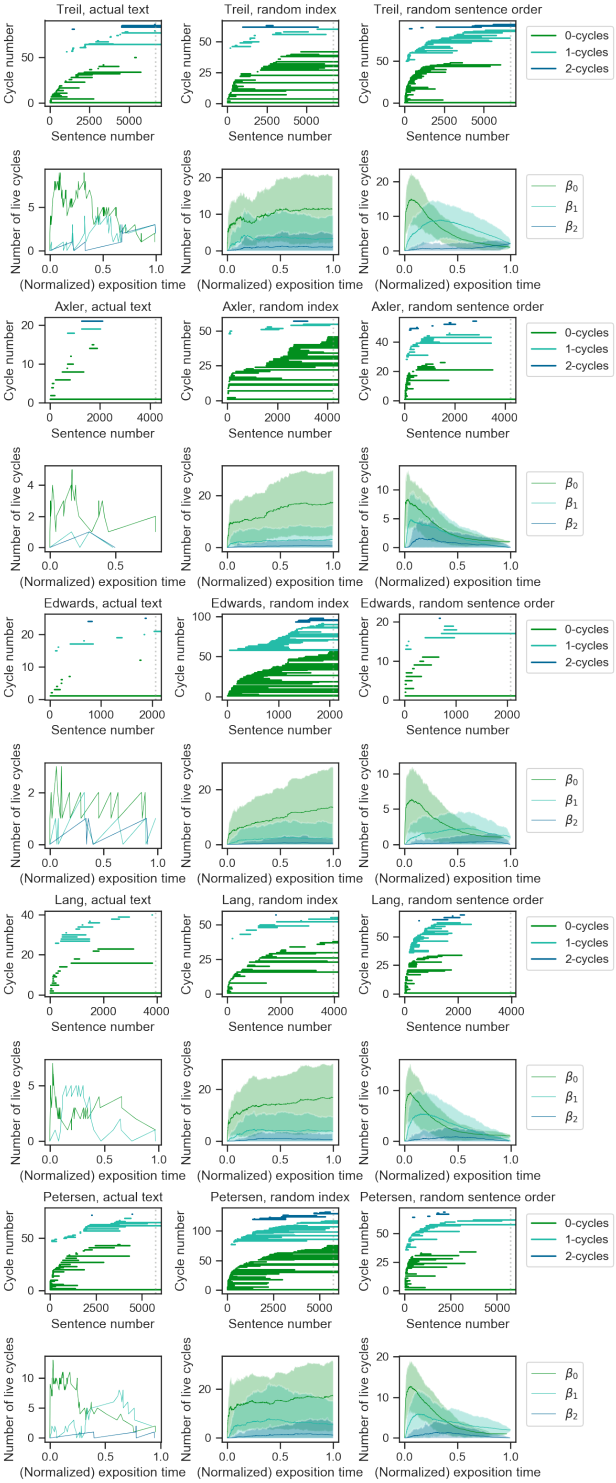

Figure S3: Sentence-filtration barcodes and Betti curves for the first half of the texts. Each pair of rows shows an example barcode and Betti curves for a given text, with text results in the leftmost column and null models in the other columns.

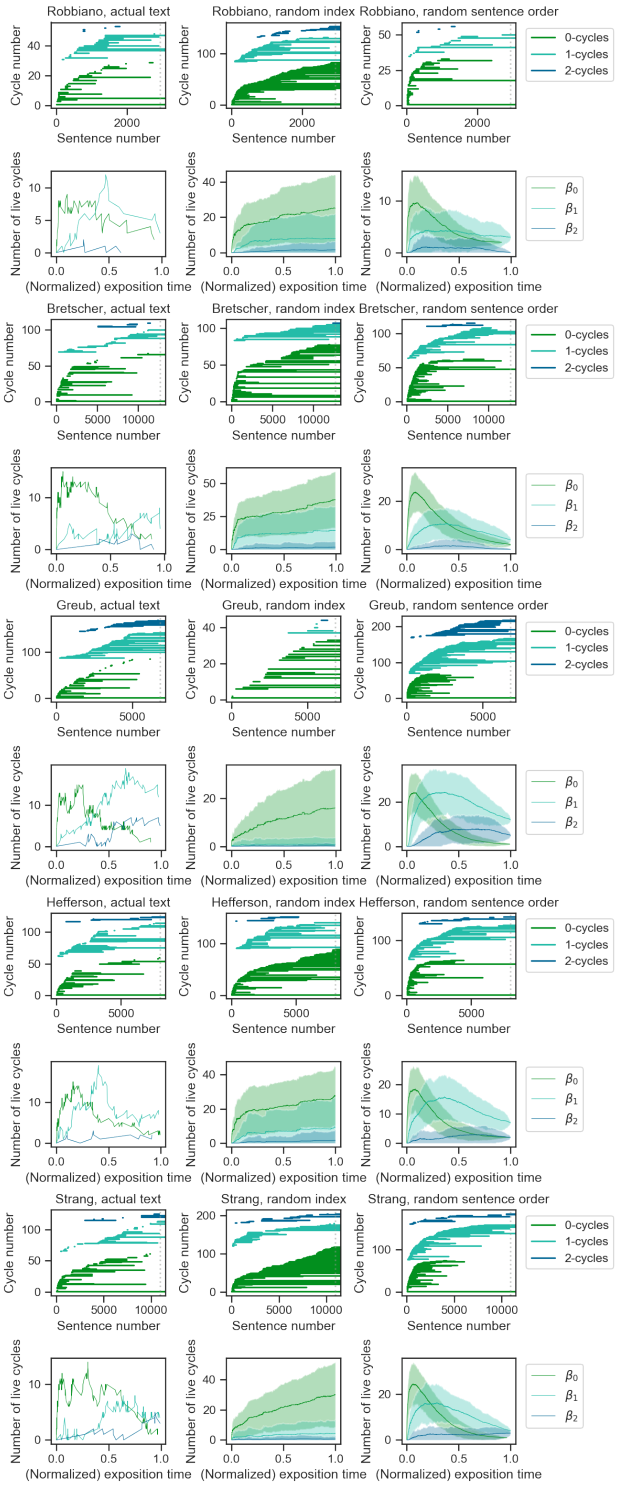

Figure S4: Sentence-filtration barcodes and Betti curves for the second half of the texts. Each pair of rows shows an example barcode and Betti curves for a given text, with text results in the leftmost column and null models in the other columns.

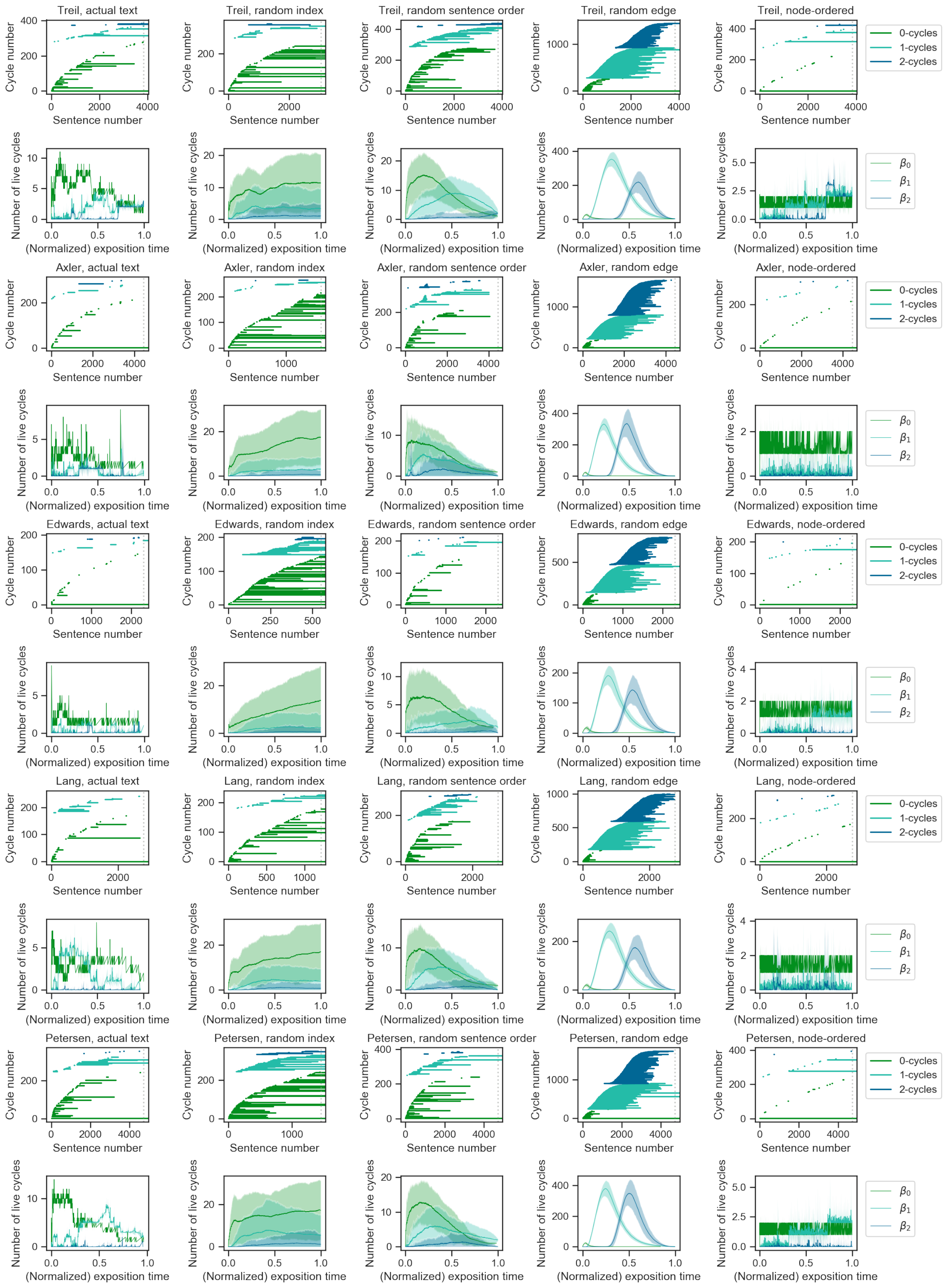

Figure S5: OAAT barcodes and Betti curves for the first half of the texts. Each pair of rows shows an example barcode and Betti curves for a given text, with text results in the leftmost column and null models in the other columns.

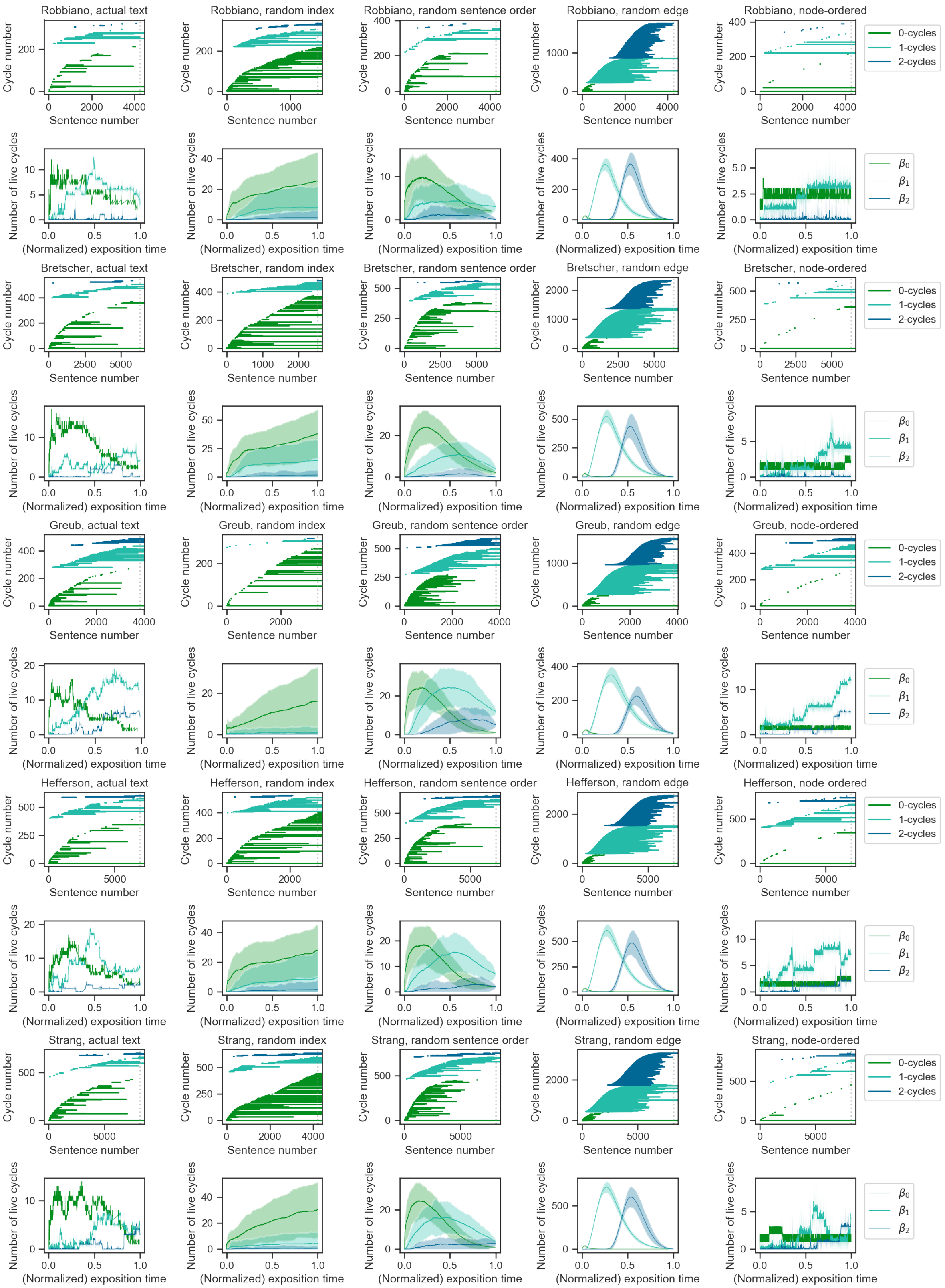

Figure S6: OAAT barcodes and Betti curves for the second half of the texts. Each pair of rows shows an example barcode and Betti curves for a given text, with text results in the leftmost column and null models in the other columns.

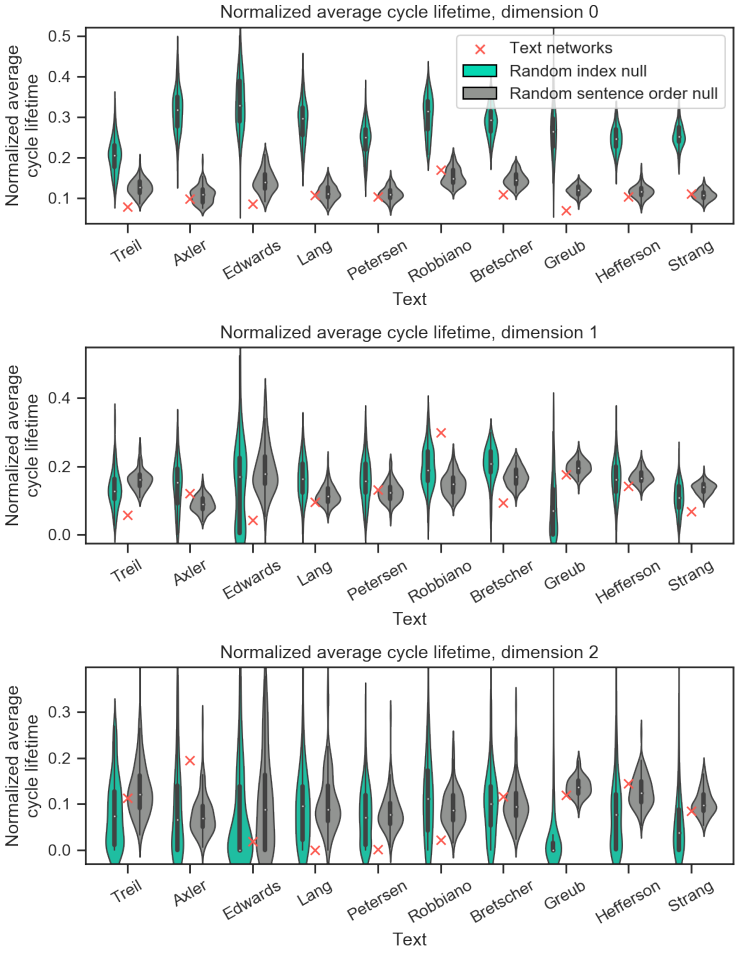

Figure S7: Normalized average cycle lifetime for 0-, 1-, and 2-dimensional persistent homology across all texts' sentence-granularity filtrations and random index and random sentence order null models. From top to bottom: dimensions 0, 1, and 2.

Text	Average Goodreads rating	Number of ratings
Treil	3.83	6
Axler	4.26	673
Lang	4.23	31
Bretscher	3.37	71
Greub	3.43	7
Hefferson	3.96	25
Strang	4.21	891

Table S6: Average rating and total number of ratings on Goodreads for texts with more than 5 ratings.

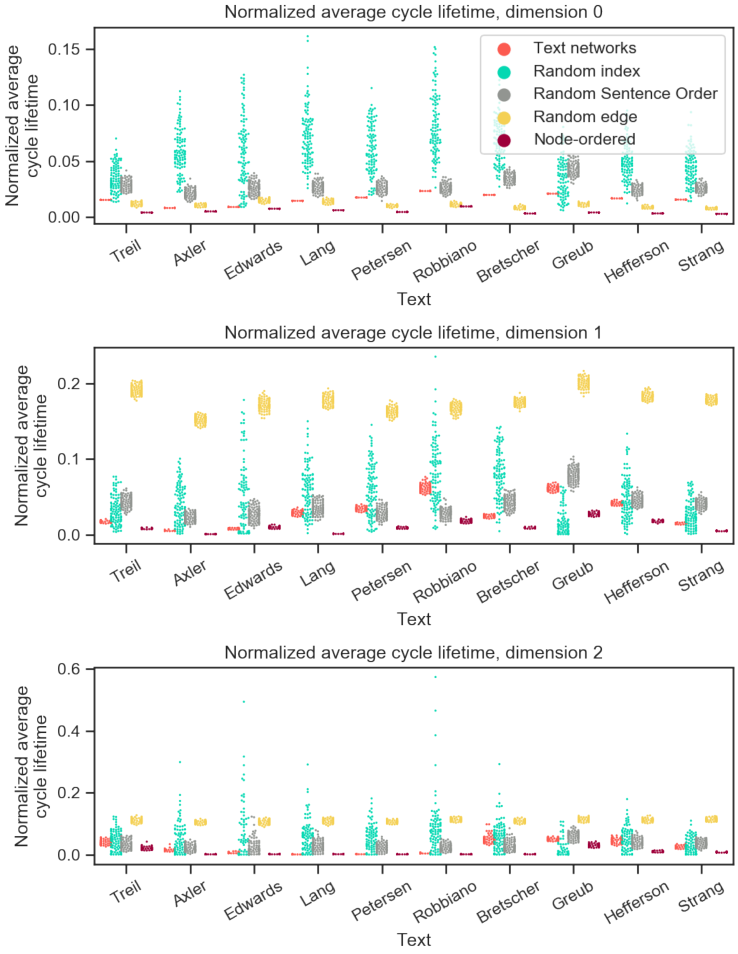

Figure S8: Normalized average cycle lifetime for 0-, 1-, and 2-dimensional persistent homology across all texts' OAAT filtrations and all null models. From top to bottom: dimensions 0, 1, and 2.

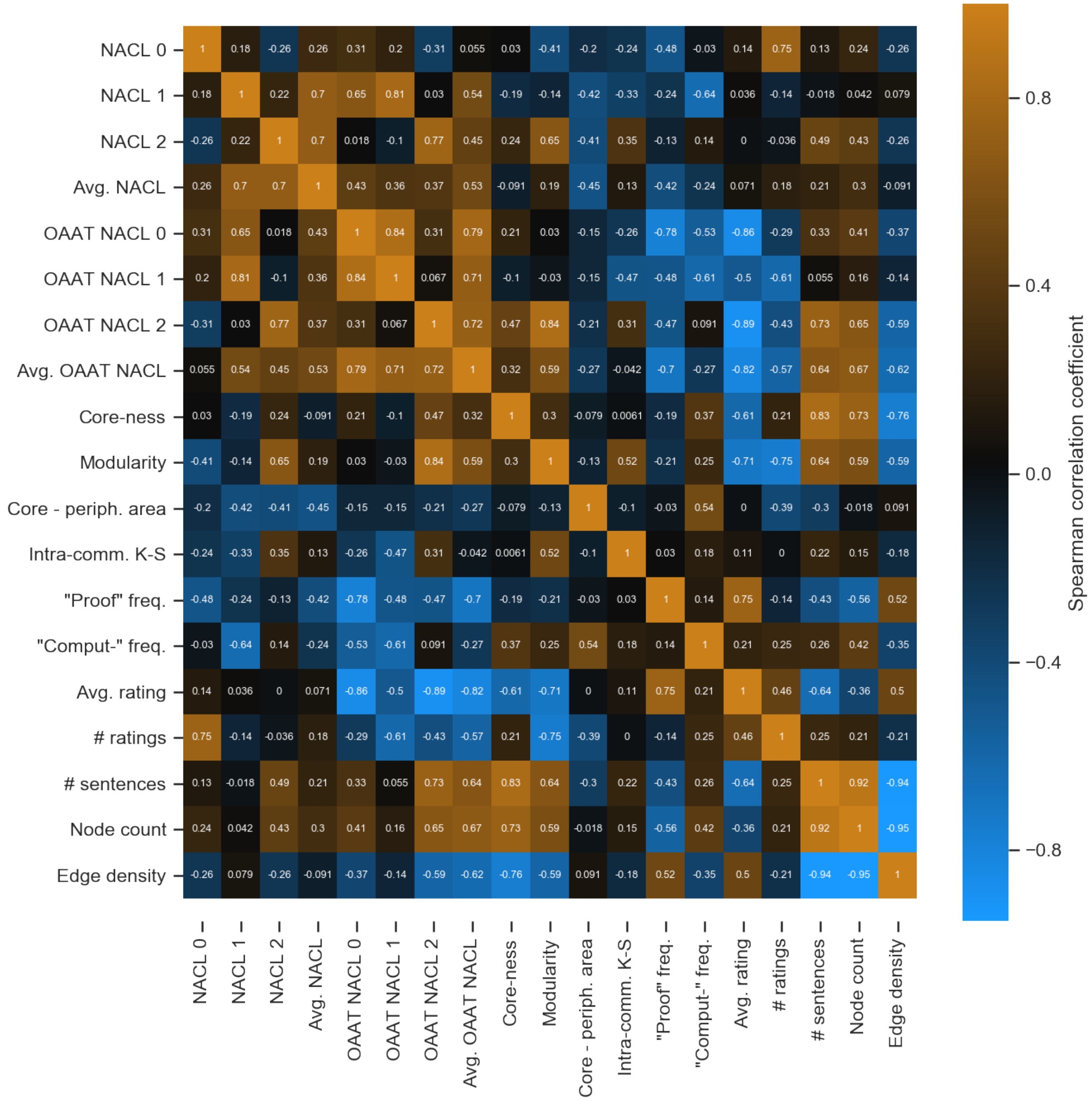

Figure S9: Spearman correlation matrix for text features, including sentence- and OAAT-normalized average cycle lifetime (NACL), core-ness and modularity statistics, core - periphery area, intra-community edge development K-S, word frequencies, average text ratings and number of ratings, and text length, node count, and edge density. “NACL d” refers to NACL in dimension d.

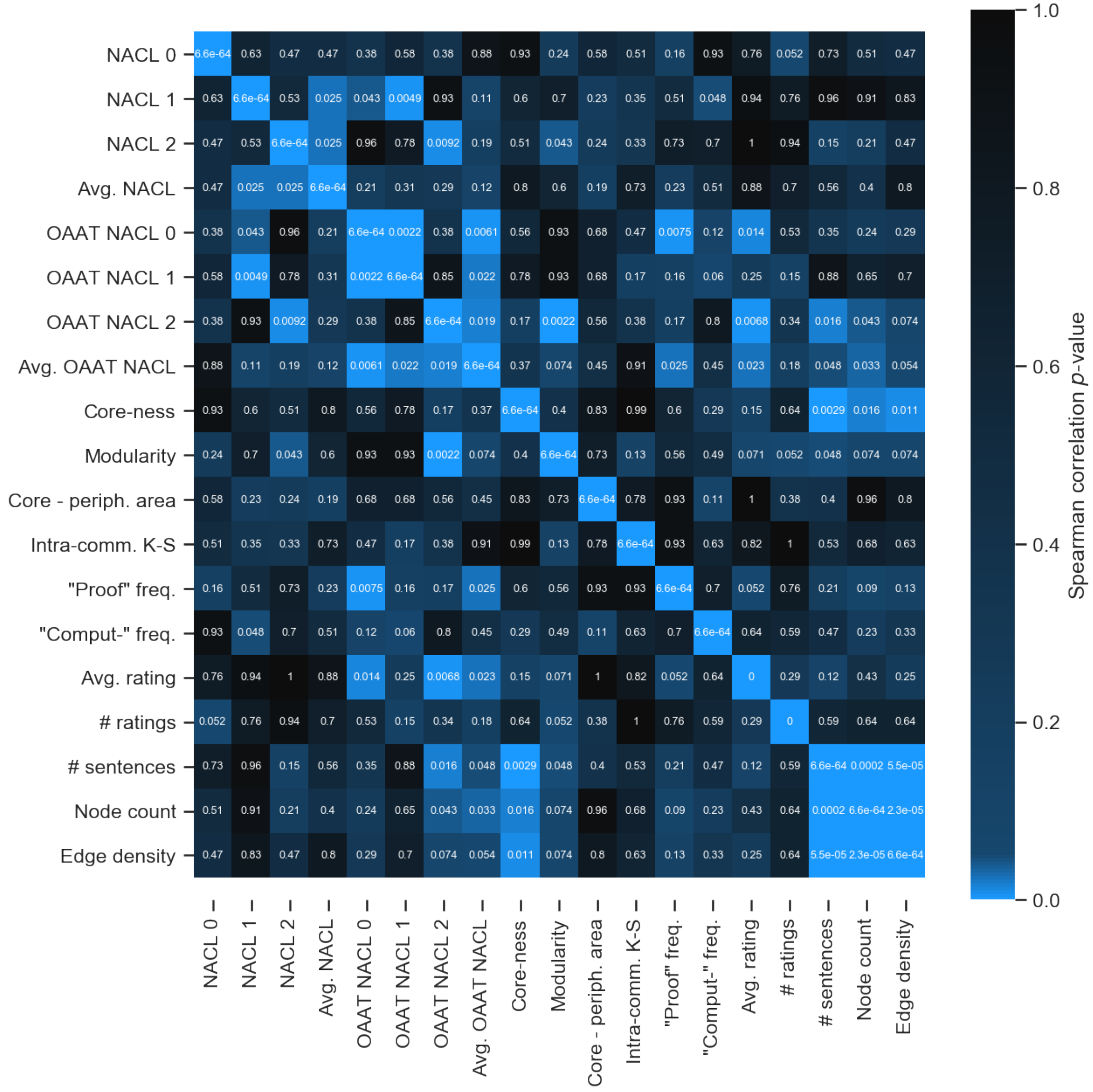

Figure S10: Spearman correlation p-values for text features, including sentence- and OAAT-normalized average cycle lifetime (NACL), core-ness and modularity statistics, core - periphery area, intra-community edge development K-S, word frequencies, average text ratings and number of ratings, and text length, node count, and edge density. “NACL d” refers to NACL in dimension d.

References

[1] Axler, S. *Linear Algebra Done Right* (Springer International Publishing, 2015), 3 edn.

[2] Bretscher, O. *Linear Algebra with Applications* (Pearson Education, Inc., 2013), 5 edn.

[3] Edwards, H. M. *Linear Algebra* (Springer Science+Business Media, 1995).

[4] Greub, W. H. *Linear Algebra* (Springer Science+Business Media, 1967), 3 edn.

[5] Hefferson, J. *Linear Algebra* (2017), 3 edn. URL `http://joshua.smcvt.edu/linearalgebra`.

[6] Lang, S. *Introduction to Linear Algebra* (Springer Science+Business Media, 1986), 2 edn.

[7] Petersen, P. *Linear Algebra* (Springer Science+Business Media, 2012).

[8] Robbiano, L. *Linear Algebra for everyone* (Springer-Verlag Italia, 2011).

[9] Strang, G. *Linear Algebra and Its Applications* (Thomson Learning, 2006), 4 edn.

[10] Treil, S. Linear algebra done wrong (2017). URL `https://www.math.brown.edu/~treil/papers/LADW/LADW.html`.

[11] Chai, L. R., Zhou, D. & Bassett, D. S. Evolution of semantic networks in biomedical texts. *J. Complex Netw.* **8** (2020). URL `http://academic.oup.com/comnet/article/8/1/cnz023/5523024`.

[12] Pereira, H., Fadigas, I., Senna, V. & Moret, M. Semantic networks based on titles of scientific papers. *Physica A: Statistical Mechanics and its Applications* **390**, 1192 – 1197 (2011).

[13] Rose, S., Engel, D., Cramer, N. & Cowley, W. Automatic keyword extraction from individual documents. In Berry, M. & Kogan, J. (eds.) *Text Mining: Applications and Theory*, chap. 1, 1 – 20 (John Wiley & Sons, Ltd, 2010).

[14] Mihalcea, R. & Tarau, P. TextRank: Bringing order into text. In *Proceedings of EMNLP 2004*, 404–411 (Association for Computational Linguistics, Barcelona, Spain, 2004). URL `https://www.aclweb.org/anthology/W04-3252`.

[15] Coulet, A., Shah, N. H., Garten, Y., Musen, M. & Altman, R. B. Using text to build semantic networks for pharmacogenomics. *J. Biomed. Inform.* **43**, 1009 – 1019 (2010). URL `http://www.sciencedirect.com/science/article/pii/S1532046410001164`.

[16] Gábor, K. *et al.* SemEval-2018 task 7: Semantic relation extraction and classification in scientific papers. In *Proceedings of The 12th International Workshop on Semantic Evaluation*, 679–688 (Association for Computational Linguistics, New Orleans, Louisiana, 2018). URL `https://www.aclweb.org/anthology/S18-1111`.

[17] Wermter, J. & Hahn, U. You can't beat frequency (unless you use linguistic knowledge): A qualitative evaluation of association measures for collocation and term extraction. In *Proceedings of the 21st International Conference on Computational Linguistics and the 44th Annual Meeting of the Association for Computational Linguistics*, ACL-44, 785–792 (Association for Computational Linguistics, Stroudsburg, PA, USA, 2006). URL `https://doi.org/10.3115/1220175.1220274`.

[18] Schütze, H. Word space. In *Adv. Neural Inf. Process. Syst. 5*, 895–902 (1993).

[19] Mcdonald, S. & Lowe, W. Modelling functional priming and the associative boost. In *Proceedings of the 20th Annual Meeting of the Cognitive Science Society*, 675–680 (Erlbaum, 1998).

[20] Bullinaria, J. A. & Levy, J. P. Extracting semantic representations from word co-occurrence statistics: A computational study. *Behav. Res. Methods* **39**, 510–526 (2007). URL `https://doi.org/10.3758/BF03193020`.

[21] Palowitch, J., Bhamidi, S. & Nobel, A. B. Significance-based community detection in weighteds networks. *J. Mach. Learn. Res.* **18**, 1–48 (2018). URL `http://jmlr.org/papers/v18/17-377.html`.

[22] Sizemore, A. E., Karuza, E., Giusti, C. & Bassett, D. Knowledge gaps in the early growth of semantic feature networks. *Nat. Hum. Behav.* **2**, 682–692 (2019).

[23] Sizemore Blevins, A. & Bassett, D. S. Reorderability of node-filtered order complexes. *Phys. Rev. E* **101**, 052311 (2020). URL `https://link.aps.org/doi/10.1103/PhysRevE.101.052311`.

[24] Virtanen, P. *et al.* SciPy 1.0: Fundamental Algorithms for Scientific Computing in Python. *Nat. Methods* **17**, 261–272 (2020).

[25] Vallat, R. Pingouin: statistics in Python. *J. Open Source Softw.* **3**, 1026 (2018).
